# Risk factors of severe cases with COVID-19: a meta-analysis

**DOI:** 10.1017/S095026882000179X

**Published:** 2020-08-12

**Authors:** Mingchun Ou, Jieyun Zhu, Pan Ji, Hongyuan Li, Zhimei Zhong, Bocheng Li, Jielong Pang, Jianfeng Zhang, Xiaowen Zheng

**Affiliations:** 1 Department of Pharmacy, People's Hospital of Baise, Baise 533000, People's Republic China; 2 Department of Emergency, The Second Affiliated Hospital of Guangxi Medical University, Nanning 530007, People's Republic China

**Keywords:** Coronavirus disease 2019, critically ill, meta-analysis, risk factors, severe disease

## Abstract

Our study aimed to systematically analyse the risk factors of coronavirus disease 2019 (COVID-19) patients with severe disease. An electronic search in eight databases to identify studies describing severe or critically ill COVID-19 patients from 1 January 2020 to 3 April 2020. In the end, we meta-analysed 40 studies involving 5872 COVID-19 patients. The average age was higher in severe COVID-19 patients (weighted mean difference; WMD = 10.69, 95%CI 7.83–13.54). Patients with severe disease showed significantly lower platelet count (WMD = −18.63, 95%CI −30.86 to −6.40) and lymphocyte count (WMD = −0.35, 95%CI −0.41 to −0.30) but higher C-reactive protein (CRP; WMD = 42.7, 95%CI 31.12–54.28), lactate dehydrogenase (LDH; WMD = 137.4, 95%CI 105.5–169.3), white blood cell count（WBC), procalcitonin（PCT）, D-dimer, alanine aminotransferase (ALT), aspartate aminotransferase (AST) and creatinine（Cr）. Similarly, patients who died showed significantly higher WBC, D-dimer, ALT, AST and Cr but similar platelet count and LDH as patients who survived. These results indicate that older age, low platelet count, lymphopenia, elevated levels of LDH, ALT, AST, PCT, Cr and D-dimer are associated with severity of COVID-19 and thus could be used as early identification or even prediction of disease progression.

## Introduction

In December 2019, Wuhan, China had reported a cluster of unexplained cases of viral pneumonia. This disease was soon named as coronavirus disease 2019 (COVID-19), and determined to be caused by a novel coronavirus, the severe acute respiratory syndrome coronavirus 2 (SARS-CoV-2) [1]. In the past two months, COVID-19 has spread across the globe. According to data released by the World Health Organization (WHO), as of 10:00 on 4 April, SARS-CoV-2 had infected 207 countries, areas or territories with a total of 1 051 697 confirmed cases and 56 986 deaths worldwide [2]. The confirmed cases in America, Italy and Spain have surpassed 100 000 and the cases continue to increase rapidly in across the world [3]. It has become a serious threat to global health and a significant challenge to health care systems worldwide.

While the disease is mild or even asymptomatic in most patients, and usually self-resolves without the need for hospitalisation, there was still a certain proportion of severe cases. The treatment of severe cases was difficult and the fatality rate was high. As of 16 February, China's COVID-19 epidemic report data showed that 19.6% of patients were severe cases [4], and the fatality rate of these cases was 49% [5]. Furthermore, a study included 52 severe case patients showed that the fatality rate was as high as 61.5% [6]. Therefore, it is critical to understand and identify the risk factors for the progression of COVID-19 patients in order to help in early identification of severe cases and improving the prognosis of patients.

Two recent systematic study reviews [7,8] of COVID-19 patients indicated increased procalcitonin values that were associated with a nearly five-fold higher risk of severe infection and low platelet count was associated with increased risk of severe disease and mortality in patients with COVID-19. However, both reviews meta-analysed small samples pooled from few studies and the indicators were not comprehensive. Recently, many large-scale clinical studies have been published [9–12], but the results across these studies were not entirely consistent. In order to gain a clearer picture of the risk factors of severe COVID-19, we meta-analysed the relevant literature. The results may provide a basis for detecting or even predicting disease progression quickly enough to improve prognosis.

## Materials and methods

### Search strategy

This meta-analysis was carried out according to preferred reporting items for Meta-Analyses of Observational Studies in Epidemiology (MOOSE) statement [13]. PubMed, Web of Science, Scopus, EMbase, CNKI, WanFang Data, Chinese Biomedical Literature Database and VIP databases were electronically searched to collect clinical studies of severe or critically ill COVID-19 patients from 1 January 2020 to 3 April 2020. We also manually searched the lists of included studies to identify additional potentially eligible studies. If there were two or more studies that described the same population, only the study with the largest sample size was chosen. There was no language restriction placed in the literature search, but only literature published online were included. The following keywords were used, both separately and in combination, as part of the search strategy in each database: ‘Coronavirus’, ‘2019-nCoV’, ‘COVID-19’, ‘SARS-CoV-2’, ‘severe’, ‘critical’, ‘icu care’, ‘mechanical ventilation’, ‘intensive care unit’, ‘mortality’, ‘fatal’, ‘death’, ‘survivors’ or ‘critically ill’.

### Study Eligibility

Studies were included in the meta-analysis if they had cohort, case−control or case-series designs; if they contained patients with mild and severe disease, or survivor and death groups; the laboratory outcomes of the COVID-19 patients included in our study were the findings when they were admitted to the hospital or first visited the hospital. At the end of the follow-up, the patients were divided into mild and severe groups. We considered disease to be ‘mild’ in those patients described in the studies as having mild or moderate disease, or ‘severe’ in those patients described as having severe disease, as being admitted to the intensive care unit or as requiring mechanical ventilation. Only studies of more than 30 patients were included.

### Data extraction and quality assessment

Three reviewers independently selected literature and extracted data to an Excel database. Any disagreement was resolved by another reviewer. The titles and abstracts were first screened to identify the eligible articles, followed by a full-text review to obtain detailed information. When required, the authors were contacted directly to obtain further information and clarifications regarding their study. Data extraction included: The first author's surname and the date of publication of the article, study design, sample size, age, outcome measurement data such as laboratory findings reported in the identified papers, relevant elements of bias risk assessment.

The quality of included studies was independently evaluated by the three reviewers based on the Newcastle-Ottawa Scale (NOS) [14] guidelines. Any disagreement was resolved by another reviewer. Studies with a score greater than 6 were considered to be of high quality (total score = 9).

### Statistical analyses

Data from studies reporting continuous data as ranges or as median and interquartile ranges were converted to mean ± s.d. [15]. The weighted mean differences (WMDs) in continuous variables between patient groups were calculated, together with the associated 95% confidence intervals (CIs). All meta-analyses were performed using STATA 12 (StataCorp, TX, USA). Since all studies were gathered from the published literature and the sample size of included studies varies greatly, so a random-effects model was used. Funnel plot together with Egger's regression asymmetry test and Begg's test were used to evaluate publication bias. A two-tailed *P* < 0.05 was regarded as statistically significant.

## Results

### Literature screening and assessment

**Fig. 1.**
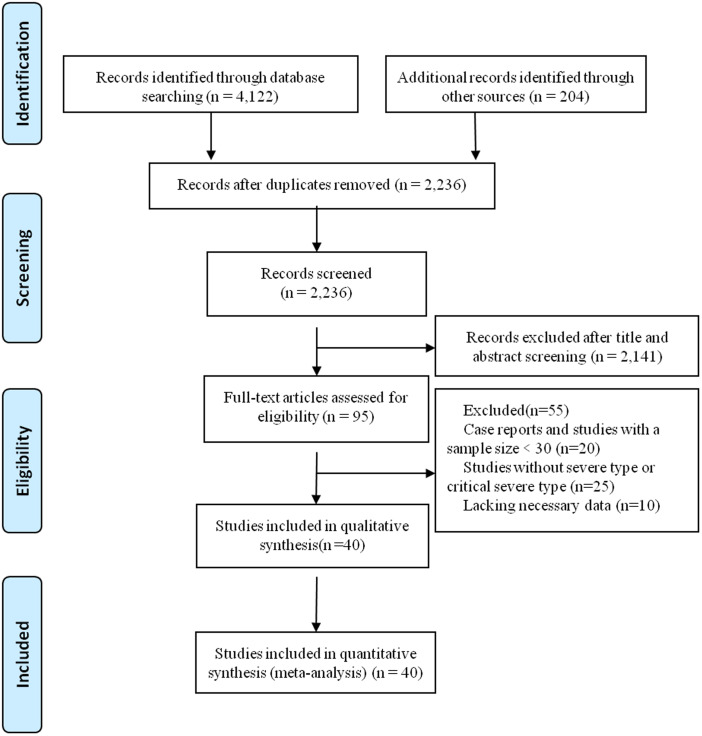
Flowchart depicting literature screening process.

A total of 4122 records were identified from the databases. In addition, 204 records were identified from the Chinese Medical Journal Network. After a detailed assessment, 40 studies [6, 9–12, 16–50] involving 5872 COVID-19 patients were included in the meta-analysis (Fig. 1).

### Characteristics of included studies

All studies included in the meta-analysis were conducted in China examined Chinese patients distributed across 31 provinces and published between 8 February 2020 and 2 April 2020. A large proportion of these studies (*n* = 37) were based on data collected from a single centre. Follow-up data were reported for most patients. All studies received quality scores of 6–9, indicating high quality (Table 1).

**Table 1.** Basic characteristics of included studies of COVID-19 patients in China

First author	Publication date in 2020	*n* (mild/severe or survival/non-survival)	Male (%)	Single- or multi-centre^a^	Study population	Age^b^, years	Follow-up	Quality score^c^
Deng [9]	20 Mar	109/116	32	Multi	Survival and non-survival COVID-19 patients	43 ± 18/68±9	1 Jan to 21 Feb	8
Zhou [16]	11 Mar	137/54	62	Multi	Survival and non-survival COVID-19 patients	56（46–67）	As of 31 Jan	8
Yang [6]	24 Feb	20/32	67	Single	Survival and non-survival COVID-19 patients	59.7–13.3	2 Dec 2019 to 23 Jan 2020	7
Chen [10]	26 Mar	161/113	62	Single	Survival and non-survival COVID-19 patients	62 (44–70)	As of 28 Feb	7
Chen [17]	12 Mar	282/181	53	Single	Mild and severe COVID-19 patients	15–90	As of 6 Feb	7
Xiao [18]	27 Feb	107/36	51	Single	Mild, severe and critically ill COVID-19 patients	45.1 ± 1.0	23 Jan to 8 Feb	9
Wang [19]	8 Feb	102/36	54	Single	Mild and severe COVID-19 patients	56（42–68）	1 Jan to 28 Jan	7
Yuan [20]	6 Mar	192/31	47	Single	Mild and severe COVID-19 patients	46.5 ± 16	24 Jan to 23 Feb	9
Fang [21]	25 Feb	55/24	57	Single	Mild and severe COVID-19 patients	45 ± 16.6	22 Jan to 18 Feb	6
Liu [22]	17 Feb	26/4	33	Single	Mild and severe COVID-19 patients	35 ± 8	10 Jan to 31 Jan	6
Zhong [23]	26 Mar	51/11	65	Single	Mild, severe and critically ill COVID-19 patients	51.8 ± 13.5	21 Jan to 10 Feb	6
Guan [24]	6 Feb	926/173	58	Multi	Mild and severe COVID-19 patients	47.0	NR	9
Qian [25]	17 Mar	82/9	42	Multi	Mild and severe COVID-19 patients	50(36.5–57)	20 Jan to 11 Feb	9
Huang [26]	15 Feb	28/13	73	Single	Mild and severe COVID-19 patients	49(41–58)	As of 2 Jan	7
LI [27]	29 Feb	58/25	53	Single	Mild and severe COVID-19 patients	45.5 ± 12.3	Jan to Feb	7
Wan [28]	21 Mar	95/40	53	Single	Mild and severe COVID-19 patients	47(36–55)	23 Jan to 8 Feb	8
Gao [29]	17 Mar	28/15	60	Single	Mild and severe COVID-19 patients	45 ± 7.7/43±14	23 Jan to 2 Feb	6
Zhang [30]	23 Feb	82/58	51	Single	Mild and severe COVID-19 patients	57.0	16 Jan to 3 Feb	7
Chen [31]	13 Mar	108/31	55	Single	Mild and severe COVID-19 patients	15–79/36–59	Jan to Feb	8
Chen [32]	17 Mar	68/21	47	Single	Mild, severe and critically ill COVID-19 patients	41.6 ± 15.6	As of 21 Feb	7
Li [33]	26 Mar	63/17	50	Single	Mild and severe COVID-19 patients	47.8 ± 19.5	20 Jan to 27 Feb	7
Li [34]	2 Apr	40/6	46	Single	Mild and severe COVID-19 patients	NR	21 Jan to 16 Feb	6
Li [35]	2 Apr	18/44	52	Single	Mild, severe and critically ill COVID-19 patients	49 ± 37/59±31	31 Jan to 25 Feb	6
Liu [11]	2 Apr	196/146	54	Single	Mild, severe and critically ill COVID-19 patients	NR	23 Jan to 12 Feb	7
Zhang [36]	2 Apr	56/18	47	Single	Mild, severe and critically ill COVID-19 patients	52.7 ± 19	21 Jan to 11 Feb	7
Xiong [37]	3 Mar	58/31	46	Single	Mild, severe and critically ill COVID-19 patients	53 ± 16.9	17 Jan to 20 Feb	7
Liu [38]	27 Mar	84/7	62	Single	Mild, severe and critically ill COVID-19 patients	NR	25 Jan to 18 Feb	6
Gao [39]	31 Mar	57/33	48	Single	Mild, severe and critically ill COVID-19 patients	51.7 ± 18.6	Jan to Feb	7
Xie [40]	2 Apr	51/28	56	Single	COVID-19 patients in Wuhan Jinyintan Hospital	60(48–66)	2 Feb to 23 Feb	7
Zhang [12]	2 Apr	84/31	40	Single	Mild and severe COVID-19 patients	43.9 ± 15/65±1	As of 22 Feb	7
Liu [41]	28 Feb	67/11	49	Multi	Mild and severe COVID-19 patients	38(33,57)	30 Dec to 15 Jan	7
Shi [42]	27 Feb	150/14	45	Single	Mild, severe and critically ill COVID-19 patients	NR	Jan to Feb	8
Shi [43]	12 Mar	38/16	57	Single	Mild, severe and critically ill COVID-19 patients	62.5 (50.5, 68.5)	9 Feb to 29 Feb	6
Peng [44]	2 Mar	96/16	47	Single	Mild and severe COVID-19 patients	62(55,67)	20 Jan to 15 Feb	7
Li [45]	20 Mar	53/13	44	Single	Mild and severe COVID-19 patients	18–82	20 Jan to 10 Feb	7
Chen [46]	27 Feb	23/25	50	Single	Mild and severe COVID-19 patients	43.8–69	24 Jan to 8 Feb	6
Wang [47]	24 Feb	132/21	50	Single	Mild and severe COVID-19 patients	43.4 ± 15/57.7±13	26 Jan to 5 Feb	8
Li [48]	5 Mar	20/10	60	Single	Mild and severe COVID-19 patients	21–72	22 Jan to 8 Feb	6
Ling [49]	18 Mar	271/21	46	Single	Mild and severe COVID-19 patients	48.7 ± 16/65.5±16	20 Jan to 10 Feb	9
Bin [50]	29 Feb	45/9	56	Single	Mild and severe COVID-19 patients	53.9 ± 17. 1	29 Jan to 16 Feb	6

a All studies were retrospective cohort studies.

b Reported as range, mean ± SD, or median (interquartile range). NR, not reported.

c Score based on the Newcastle−Ottawa scale guidelines [14].

### Meta-analysis

#### Age distribution

**Fig. 2.**
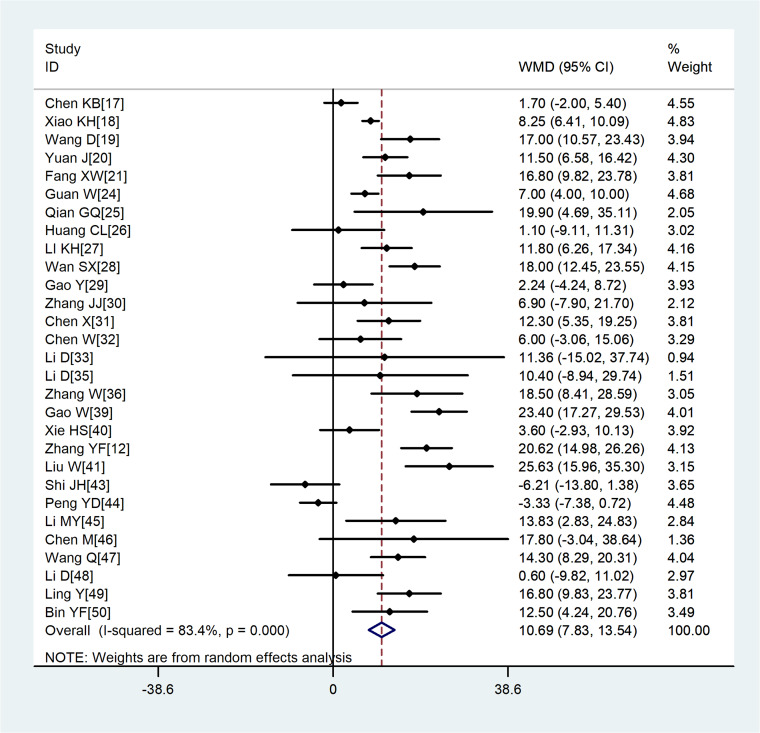
Meta-analysis of the difference in the average age between COVID-19 patients with mild or severe disease. WMD, weighted mean difference.

A total of 29 studies involving 3411 COVID-19 patients were included. Although the heterogeneity was high across enrolled studies, the result showed that compared with non-severe group, the age of severe group was higher (WMD = 10.69, 95%CI 7.83–13.54) (Fig. 2).

#### Laboratory parameters

**Fig. 3.**
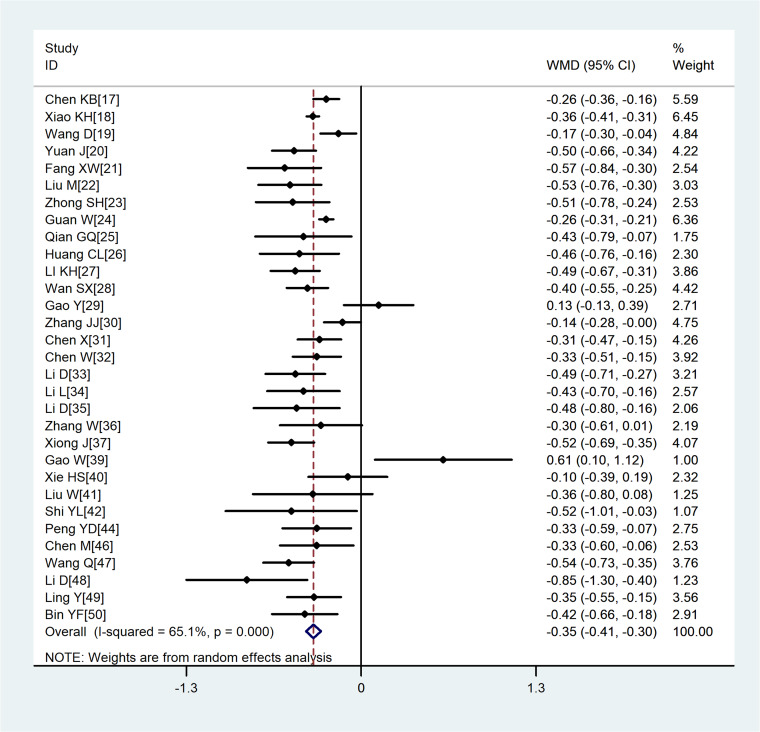
Meta-analysis of the difference in the lymphocyte count between COVID-19 patients with mild or severe disease. WMD, weighted mean difference.

**Fig. 4.**
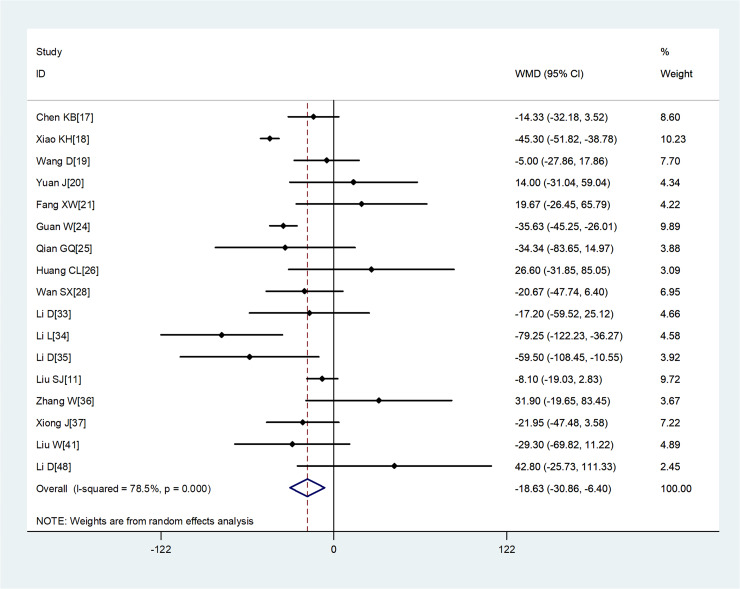
Meta-analysis of the difference in the platelet count between COVID-19 patients with mild or severe disease. WMD, weighted mean difference.

**Fig. 5.**
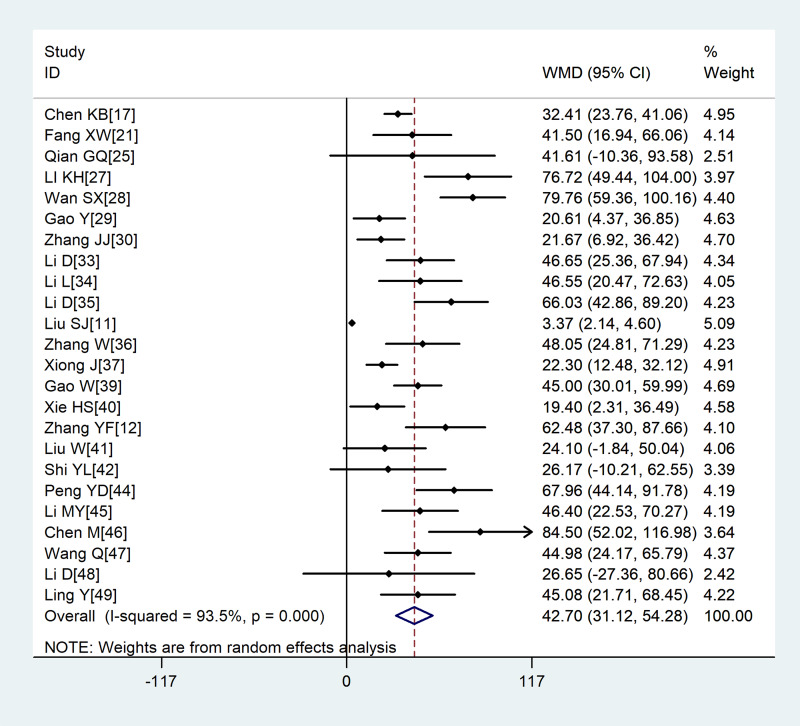
Meta-analysis of the difference in the C-reactive protein between COVID-19 patients with mild or severe disease. WMD, weighted mean difference.

**Fig. 6.**
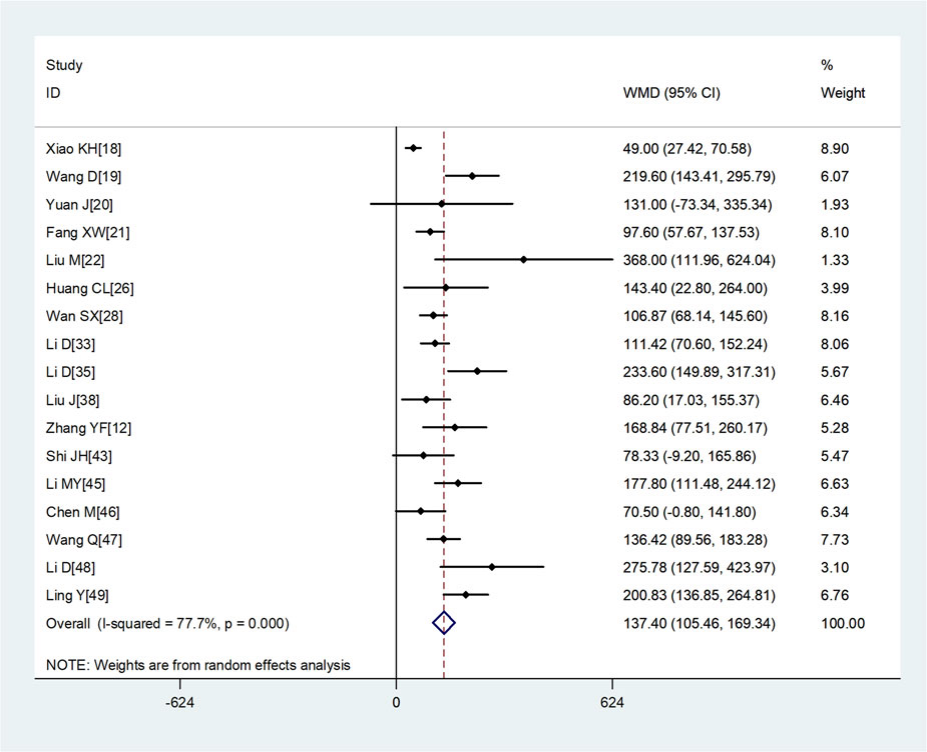
Meta-analysis of the difference in the lactate dehydrogenase between COVID-19 patients with mild or severe disease. WMD, weighted mean difference.

Compared with non-severe group, the lymphocyte count (WMD = −0.35, 95%CI −0.41 to −0.30) and the platelet count (WMD = −18.63, 95%CI −30.86 to −6.40) were found to be lower, while C-reactive protein (CRP; WMD = 42.7, 95%CI 31.12–54.28) and lactate dehydrogenase (LDH; WMD = 137.4, 95%CI 105.5–169.3) were significantly higher in the severe group (Figs 3–6). Patients in the severe group also displayed elevated levels of white blood cell count (WBC; WMD = 0.93, 95%CI 0.51–1.36), procalcitonin (PCT; WMD = 0.07, 95%CI 0.05–0.10), D-dimer (WMD = 0.38, 95%CI 0.24–0.52), alanine aminotransferase (ALT; WMD = 5.12, 95%CI 0.82–9.42), aspartate aminotransferase (AST; WMD = 8.51, 95%CI 5.01–12.01) and creatinine (Cr; WMD = 4.57, 95%CI 0.64–8.50) compared to those in the non-severe group (Table 2).

**Table 2.** Meta analysis of different laboratory parameters in COVID-19 patients

Laboratory parameters	No. studies	No. patients	Heterogeneity		Model	Meta analysis	
			*P*	*I*^2^		WMD（95%CI）	*P*
Severe *vs.* mild disease							
Age, years	29	4306	< 0.001	83.4%	Random	10.69 (7.83,13.54)	< 0.001
WBC, × 10^9^/l	32	4736	< 0.001	83.2%	Random	0.93（0.51,1.36）	< 0.001
LBC, × 10^9^/l	31	4456	< 0.001	65.1%	Random	−0.35（−0.41,−0.30）	< 0.001
PLT, × 10^9^/l	17	3211	< 0.001	78.5%	Random	−18.63（−30.86,−6.40）	0.003
PCT, ng/ml	23	3087	< 0.001	89.8%	Random	0.07（0.05,0.10）	< 0.001
D-dimer, μg/ml	18	2169	< 0.001	66.3%	Random	0.38（0.24,0.52）	< 0.001
CRP, mg/l	24	2964	< 0.001	93.5%	Random	42.7（31.12,54.28）	< 0.001
LDH, U/l	17	1792	< 0.001	77.7%	Random	137.4（105.46,169.34）	< 0.001
ALT, U/l	22	2440	< 0.001	71.0%	Random	5.12（0.82,9.42）	0.020
AST, U/l	22	2452	< 0.001	74.7%	Random	8.51（5.01,12.01）	< 0.001
Cr, μmol/ml	17	1922	0.026	61.6%	Random	4.57（0.64,8.50）	0.023
Death *vs.* survival							
Age, years	4	742	0.002	79.2%	Random	18.68 (14.15,23.21)	< 0.001
WBC, × 10^9^/l	3	690	0.024	73.3%	Random	4.14（2.87,5.41）	< 0.001
LBC, × 10^9^/l	4	742	0.188	37.4%	Random	−0.43（−0.5, −0.35）	< 0.001
PLT, × 10^9^/l	2	243	0.001	90.9%	Random	−12.94 (−92.78,66.89)	0.751
D-dimer, μg/ml	2	465	0.881	0.0%	Random	8.34 (6.14,10.64)	< 0.001
LDH, U/l	2	465	< 0.001	97.6%	Random	139.3（−188.05,466.7）	0.404
ALT, U/l	3	690	0.033	70.6%	Random	7.23（2.25,12.2）	0.004
AST, U/l	2	499	0.003	88.6%	Random	16.68 (7.48,25.89)	< 0.001

CI, confidence interval; WMD, weighted mean difference.

Four studies [6, 9, 10, 16] whose primary outcome was death were also analysed. The results showed that on admission, patients who died showed significantly higher WBC, D-dimer, ALT, AST and Cr but similar platelet count and LDH as patients who survived (Table 2).

#### Sensitivity analysis

**Fig. 7.**
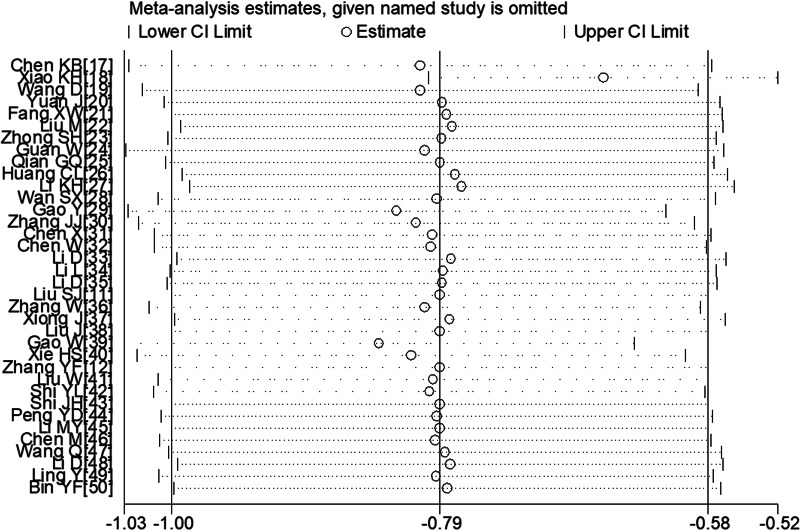
Sensitivity analysis of the lymphocyte count between COVID-19 patients with or without severe disease.

To determine sensitivity, we removed each study one by one and the pooled results did not change substantially, indicating the reliability and stability of our meta-analysis (e.g. Figure 7).

### Publication bias

**Fig. 8.**
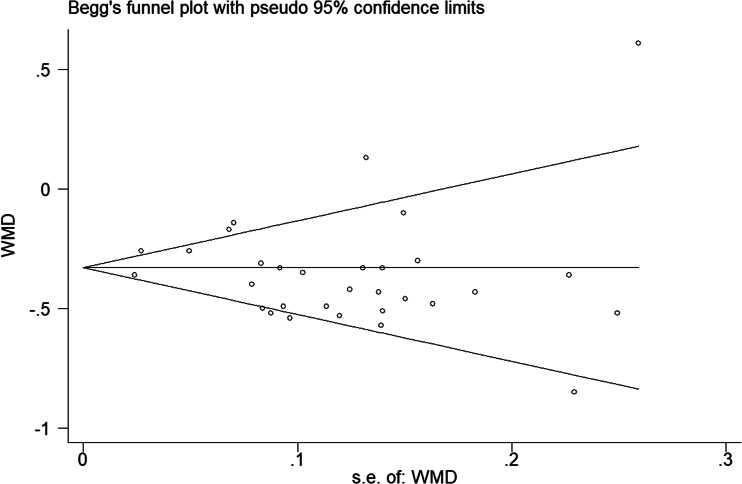
Funnel plot regarding the outcome of lymphocyte count.

The *P* values derived using the Egger's and the Begg's test for all outcomes showed no obvious publication bias (Table 3). A funnel plot based on the outcome of lymphocyte count showed the *P*-values of Egger's and Begg's test were 0.315 and 0.919, respectively, indicating that the publication bias did not exist (Fig. 8).

**Table 3.** Evaluation of publication bias using the Egger's and the Begg's test

Group	Age	WBC	LBC	PLT	PCT	D-dimer	CRP	LDH	ALT	AST	Cr
*P*-values of Egger's test	0.167	< 0.001	0.315	0.035	< 0.001	0.072	< 0.001	0.001	0.026	0.009	0.371
*P*-values of Begg's test	0.985	0.062	0.919	0.484	0.792	0.049	0.264	0.232	0.091	0.236	0.387

## Discussion

In this study, we meta-analysed the relevant literature from 1 January 2020. Our analysis of 40 studies [9–12, 15–50] involving 5872 COVID-19 patients suggests that lymphocyte and platelet count were found to be lower in those with severe disease than in those with mild disease, and significantly lower in those who die during follow-up than in those who survive. One plausible explanation is severely impaired immune function in severe cases, accompanied by lymphocyte necrosis and apoptosis, resulting in decreased lymphocytes in peripheral blood. According to the study by Zarychanski *et al*. [51], thrombocytopenia was commonplace in severe or critically ill patients, and usually suggests serious organ malfunction and may evolve towards disseminated intravascular coagulation (DIC).

We also found that LDH, ALT, AST and Cr were higher in severe or death group, which suggested that the heart, liver, kidney and other important organ functions were more severely damaged in severe patients. Studies have shown that elevated levels of LDH was a risk factor for mild patients progressing to become critically ill patients [52] and the incidence of myocardial injury was greater in severe patients [45]. A recent meta-analysis included 341 COVID-19 patients, and the results showed that the values of cTnI were found to be significantly increased in COVID-19 patients with severe disease than in those without (SMD = 25.6, 95% CI 6.8–44.5) [53]. According to Xie *et al*. [40], liver injury was common in hospitalised COVID-19 patients, and it may be related to systemic inflammation. Therefore, intense monitoring and evaluation of liver function in COVID-19 patients should be considered. In addition, PCT and CRP were higher in the severe cases of this study. Since the production and release into the circulation of PCT from extrathyroidal sources is enormously amplified during bacterial infections [7], suggesting that severe cases were more likely to have a bacterial infection, so serial PCT measurement may play a role for predicting evolution towards a more severe form of the disease.

According to the study by Mahase [54], the overall fatality rate in COVID-19 patients has been estimated at 0.66%, rising sharply to 7.8% in people aged over 80 and declining to 0.0016% in children aged 9 and under. In Italy, the case-fatality rate even reached 20.2% in people aged over 80 [55]. In our study, severe patients were older compared to non-severe patients. These results suggest that older age is associated with an increased risk of death. The underlying reasons may be that older age had a more significant number of comorbid conditions such as hypertension and diabetes mellitus, most of the chronic diseases share several standard features with infectious disorders, such as the proinflammatory state, and the attenuation of the innate immune response. Therefore, older age and comorbidities could be risk factors for severe patients.

Although our meta-analysis rigorously analysed data from a large sample of COVID-19 patients, our results are limited by the heterogeneity observed across studies. For example, given that most of the studies included in our meta-analysis were single-centre, retrospective studies, it was difficult for us to control for the effects of several confounding factors, including the disease course and severity, the participants' inclusion criteria as well as the studies design. Additionally, the studies included in our meta-analysis were from China, not those infected in other countries, so geographical and ethnic differences were not excluded whether the conclusion was consistent in other countries needs to be further investigated.

## Conclusion

In summary, current evidence showed that, older age, low platelet count, lymphopenia, elevated levels of LDH, ALT, AST, PCT, Cr and D-dimer were associated with severity of COVID-19. And thus could be used as early identification or even prediction of worsening illness. Due to the limited quality and quantity of the included studies, more high-quality prospective studies are required to verify the above conclusions.

## Data Availability

The data that support the findings of this study are available from the corresponding author upon reasonable request.
